# Long-Term Denosumab Treatment in Adults with Juvenile Paget Disease

**DOI:** 10.1007/s00223-025-01370-0

**Published:** 2025-04-13

**Authors:** Stergios A. Polyzos, Konstantinos Anastasilakis, Tim Cundy, Marina Kita

**Affiliations:** 1 First Laboratory of Pharmacology, School of Medicine, Aristotle University of Thessaloniki, Thessaloniki, Greece; 2 Department of Ophthalmology, 424 General Military Hospital, Thessaloniki, Greece; 3 Faculty of Medical & Health Sciences, University of Auckland, Auckland, New Zealand; 4 Department of Endocrinology, Ippokration General Hospital, Thessaloniki, Greece

**Keywords:** Denosumab, Juvenile Paget disease, Osteoprotegerin, RANK, RANKL, *TNFRSF11B* gene

## Abstract

Juvenile Paget disease (JPD) is a very rare disease, mainly caused by biallelic inactivating mutations in the *TNFRSF11B* gene that encodes osteoprotegerin. Owing to its rarity, the treatment of JPD is largely empirical. Accelerated bone turnover as assessed by biochemical markers, such as alkaline phosphatase (ALP), can be suppressed by bisphosphonate treatment, but it relapses if bisphosphonate treatment is discontinued. In this report, we describe our experience with long-term denosumab treatment in two adults with JPD, homozygous for the “Balkan” mutation (966_969delTGACinsCTT) in *TNFRSF11B.* Subject 1 started denosumab in age 35 and subject 2 in age 34. Both continue treatment until today, for 13.5 and 12 years, respectively. ALP was steadily normalized in both. Bone pain decreased and mobility improved. Hearing did not further deteriorate and no new fracture occurred. Vision remained unchanged in subject 2, but subject 1 experienced sudden vision loss of the right eye at age 46, which was successfully managed with intravitreal treatment with anti-vascular endothelial growth factor medications. In conclusion, long-term denosumab administration in adults with JPD, who had been previously treated with bisphosphonates, was safe and effective in terms of the skeletal disease, but it may not prevent the emergence of retinopathy.

## Background

Juvenile Paget disease (JPD, MIM # 239000) is mainly caused by biallelic inactivating mutations in the *TNFRSF11B* gene that encodes osteoprotegerin (OPG) [1, 2], although mutations in other genes, including *TNFRSF11A* encoding receptor activator of nuclear factor-κB (RANK) [3] and *specificity protein* *7* (*SP7*) encoding osterix [4], have been causally linked with JPD [5]. Osteoclast activity is driven by the interaction of the RANK ligand (RANKL), secreted by osteoblasts, with its receptor on osteoclasts, RANK [6]. OPG, also secreted by osteoblasts, acts as a decoy receptor for RANKL, so dampening down bone resorption [6]. Loss of OPG action causes generalized, rapid bone turnover with elevated biochemical markers of turnover [5]. In childhood, skeletal manifestations dominate (short stature, bone pain, deformity, fractures, macrocephaly and deafness (caused by destruction of the inner ear bones), but with aging, extra-skeletal manifestations appear, including retinopathy, vascular calcification and aneurysm formation, most commonly in the internal carotid and iliac arteries [5, 7, 8]. OPG deficient mice also develop vascular calcification and, in recognition of this, it has been suggested that osteoprotegerin might better be termed “osteovasculoprotegerin” [9].

The severity of the skeletal phenotype seems to be associated to the nature of the *TNFRSF11B* gene mutation [5]. The so-called “Balkan” mutation (966_969delTGACinsCTT), previously identified in five people [10–14], is associated with a relatively mild skeletal phenotype.

Owing to its rarity, the treatment of JPD is largely empirical [5]. Accelerated bone turnover as assessed by biochemical markers, such as alkaline phosphatase (ALP), can be suppressed by bisphosphonate treatment, but accelerated bone turnover relapses, if bisphosphonate treatment is discontinued [5]. If applied intensively and early in life, bisphosphonates may ameliorate most skeletal manifestations of JPD [15], but they do not seem to impact the extraskeletal manifestations [5, 10]. Replacement therapy with recombinant OPG has favorable medium-term effects (15 months) on skeletal disease [16], but it is not commercially available, nor is likely to become so [17].

Denosumab, is a monoclonal antibody against RANKL that prevents RANKL from binding to and activating RANK. Denosumab is widely used in the treatment of various types of osteoporosis and other rarer metabolic bone diseases, in which the RANKL/RANK/OPG system is disordered [18]. To date only two short-term studies have described the use of denosumab in JPD [11, 19]. In this report we describe our experience with long-term denosumab treatment in two adults with JPD, homozygous for the “Balkan” mutation in *TNFRSF11B*.

## Methods

Denosumab administration was approved by the scientific and ethics committee of Ippokration General Hospital, Thessaloniki, Greece, and the report was also approved by the Bioethics committee of the School of Medicine, Aristotle University of Thessaloniki, Greece. Signed informed consent was obtained from both patients. All procedures were in accordance with the principles of good clinical practice and the declaration of Helsinki. Biochemical parameters of interest, including serum ALP and calcium, were measured by standard methods using an automated analyzer (Olympus AU2700; Olympus, Hamburg, Germany). A 10-point visual analog scale (VAS) was used to estimate bone pain, as previously reported [20]. Audiography and fundoscopy, and more recently optical coherence tomography were undertaken periodically.

## Case Presentation

Subject 1 born in 1976 and has been previously described [11, 21]. Skeletal deformity and growth retardation were evident at the age of four years. Her peak height was 151 cm. She had marked macrocephaly with bowed long bones, kyphoscoliosis and a right femur fracture at age 9. Impaired hearing was first evident at adolescence and retinal angioid streaks at the age of 33 years. Off treatment, ALP was 1300–2500 IU/l (reference range 25–120 IU/l). She had received in the past multiple, intermittent courses of calcitonin, etidronate and risedronate from age 8, which led to incomplete suppression of ALP and resurgence soon after treatment discontinuation (nadir ALP 684 IU/l after risedronate). Zoledronate was administered twice (age 31 and 34 years). She developed profound hypocalcemia due to hungry bone syndrome after the first infusion [21], but she had a better biochemical response compared with previous bisphosphonate treatment (nadir ALP 400 IU/l and 140 IU/l, respectively).

Subject 2 born in 1979 and has been previously reported [14]. Mild skeletal deformity was evident from childhood, but without growth retardation (peak height 177 cm). He has narrowing of the upper half thoracic cage, bowing of the long bones and he experienced low-energy fractures of both humeri, both clavicles, right knee and left femur between the ages of 4 and 20. Impaired hearing was first evident at adolescence and retinal angioid streaks at the age of 32 years. Arterial hypertension was evident from age 25, being well controlled under treatment with zofenopril and bisoprolol. Off treatment, his ALP was 630–800 IU/l. He had been treated with multiple, intermittent courses of calcitonin, alendronate and risedronate from age 8, the latter leading to near normalization of ALP (nadir 132 IU/l), which again rose after treatment discontinuation. Zoledronate was administered twice (age 27 and 30 years) leading to normalization of ALP (nadir 101 and 95 IU/L, respectively) after treatment, with a subsequent slow increase.

Subject 1 started denosumab at age 35 and subject 2 at age 34. Both continue treatment (to date 13.5 and 12 years, respectively). The dose and frequency of denosumab administration was guided by the tendency of ALP to climb and by the reappearance of bone pain, which decreased after each denosumab injection [11]. Stable doses of 30 mg denosumab were established (0.58 mg/kg body weight every 3 months and 0.35 mg/kg every 2½ months, respectively). Both also received oral calcium and vitamin D as needed.

**Fig. 1:**
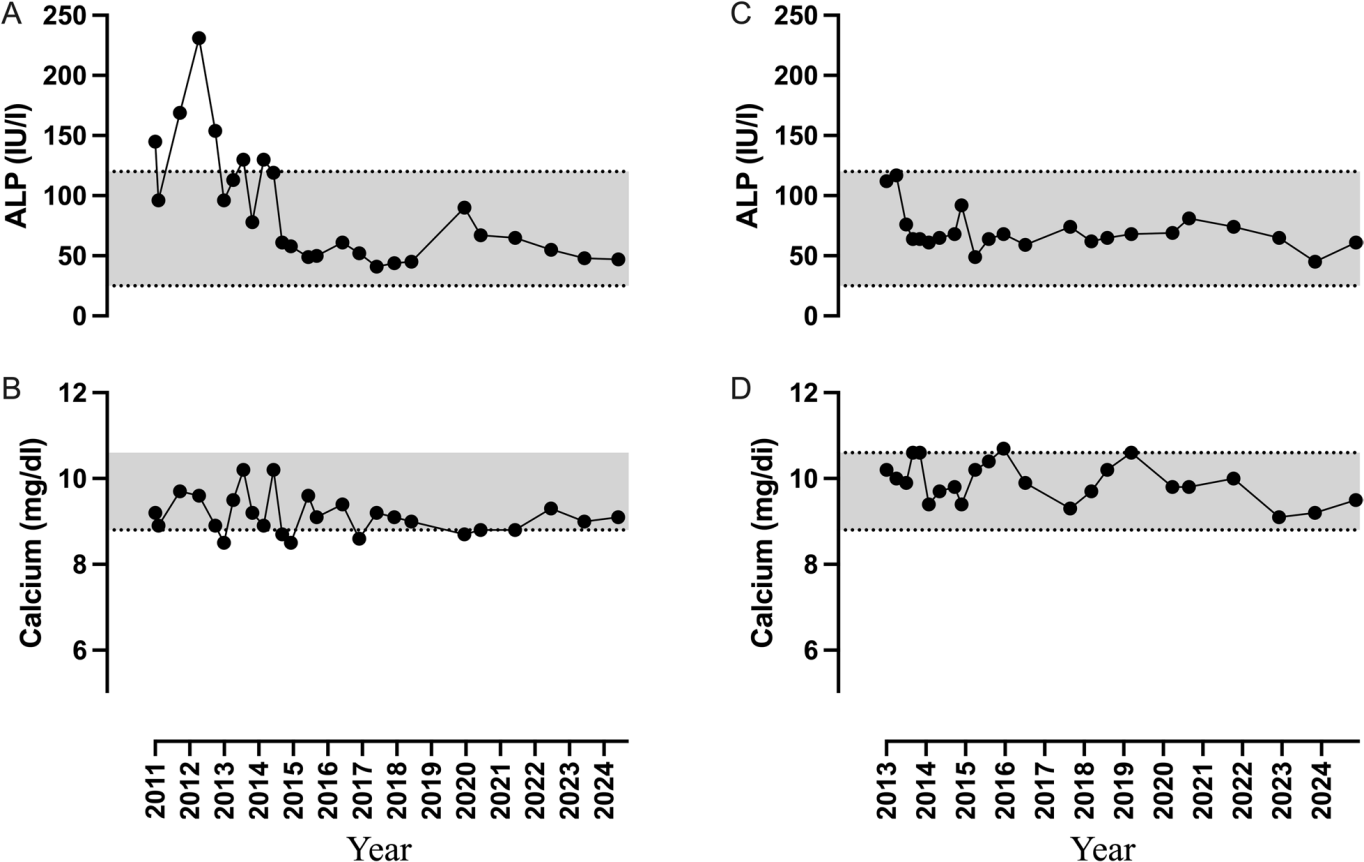
Changes in serum ALP and calcium during the long-term denosumab administration in subject 1 (**A** and **B**, respectively) and subject 2 (**C** and **D**, respectively). The grey shadow indicates the reference range of ALP (25–120 IU/l) and calcium (8.8–10.6 mg/dl). *ALP* Total Alkaline Phosphatase

The effect of the first 2 years of denosumab administration in subject 1, was described in our previous report [11]. ALP was steadily normalized in both (Fig. 1). No new fractures occurred, bone pain decreased and mobility improved. The VAS for bone pain was 9/10 and 7/10 before denosumab in subject 1 and 2, respectively, and it varied in both from 0–2/10 one-two weeks after each denosumab injection to 2–5/10 before each next denosumab injection.

Neither subject reported deterioration in hearing. Audiography was undertaken before starting denosumab: subject 1 showed moderate (lower frequencies) to moderately severe (higher frequencies) hearing loss of both ears, which was predominantly conductive; subject 2 showed moderate (lower frequencies) to moderately severe (higher frequencies) mixed hearing loss of both ears. After 13 and 11.5 years, respectively, of denosumab treatment, repeat audiography showed no significant change.

**Fig. 2:**
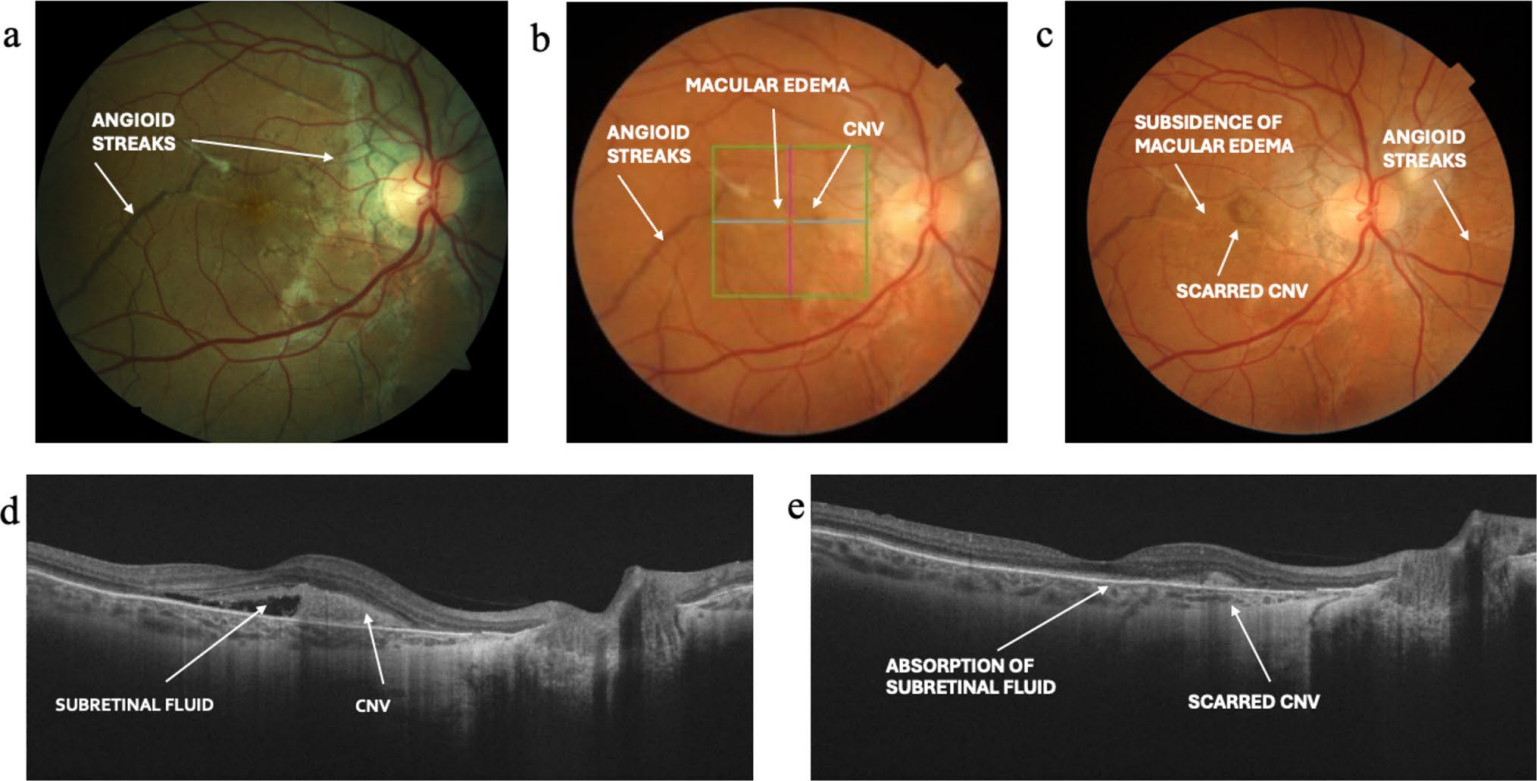
Deterioration of retinopathy of the right eye in subject 1, before and after intravitreal treatment with anti-VEGF. **a** Fundus photo taken after 4 years’ denosumab treatment, indicating angioid streaks (visual acuity 9/10; age 39); **b** fundus photo indicating angioid streaks, macular edema and CNV before intravitreal treatment with anti-VEGF (visual acuity 1/10; age 46); **c** fundus photo indicating angioid streaks, subsidence of macular edema and scarred CNV after treatment with anti-VEGF (visual acuity 8/10; age 47); **d** OCT indicating subretinal fluid and adjacent CNV before treatment with anti-VEGF (age 46); **e** OCT indicating complete absorption of subretinal fluid and scarred CNV after treatment with anti-VEGF (age 47). *CNV* Choroidal Neovascularization, *OCT* Optical Coherence Tomography *VEGF* Vascular Endothelial Growth Factor

Vision remained unchanged in subject 2, but subject 1 experienced sudden vision loss of the right eye at age 46. Examination showed macular edema, subretinal fluid and adjacent choroidal neovascularization. She had almost full recovery of vision following intravitreal treatment with bevacizumab (once) and aflibercept (four times) (Fig. 2).

Denosumab was well-tolerated. Asymptomatic hypocalcemia after each denosumab injection was evident in subject 1 during the first 2 years of treatment, but plasma calcium remained within the normal range thereafter (Fig. 1).

## Discussion

JPD is very rare, so understanding of its optimal treatment must for now rely on learning from individual case studies. Our report describes two adults with JPD in whom long-term denosumab treatment provided excellent control of the accelerated bone turnover, without any safety concerns. We arrived empirically at doses and intervals that controlled both biochemical marker and symptoms, without inducing clinically significant hypocalcemia. These doses were lower (0.58 and 0.35 mg/kg body weight) than used in adult osteoporosis (typically 0.6 to 1.0 mg/kg), but were required more frequently, every 10 to 13 weeks, rather than 6 monthly [18].

While denosumab was able to normalize bone turnover, it is not clear whether it is more effective than bisphosphonate treatment. Bone turnover is higher in children than in adults and to control accelerated bone turnover in children with JPD, bisphosphonate treatment needs to be given in doses higher than those used in other disorders, such as osteogenesis imperfecta. It now seems clear that treatment should be continuous at least until growth is complete [11, 15]. The re-emergence of bone pain between denosumab injections in the adults we studied suggests that continuous lifetime treatment is needed.

Adequate suppression of bone turnover is important in children with JPD to minimize the development of skeletal deformity [15]. Profound hypocalcemia, i.e., “hungry bone syndrome”, can ensue, if either bisphosphonates or denosumab are given when bone turnover is very high [19, 21]. We did not observe this phenomenon in the cases described here, because bone turnover was already relatively low at the time denosumab was started, in part because of previous bisphosphonate therapy and in part because they were adults.

To our knowledge only one child with JPD has had a trial of denosumab and the case illustrates its potential hazards. This child was treated with pamidronate from the age of 3½ to 6½ years, with incomplete reduction of bone turnover to age appropriate values. At age 8, she was given denosumab (0.5 mg/kg body weight) with a rapid and profound reduction in bone resorption with severe hungry bone syndrome [19]. A second dose (0.5 mg/kg body weight) was given seven weeks later in response to worsening bone pain and increasing bone turnover. The duration of response was short-lived, with a rapid increase in bone resorption and the development of severe hypercalcemia [19]. Hypercalcemia after withdrawal of denosumab is much more common in children than adults, emphasizing that “rebound” high bone resorption rates are critically associated with this phenomenon [22]. Since “hungry bone syndrome” may be evident in children with JPD after either bisphosphonate or denosumab [19, 21], a relatively low dose of antiresorptives should be used to initiate treatment, and progressively increased as bone turnover is decreased [5]. Particularly in children, a bisphosphonate would appear safer than denosumab for treatment initiation.

A distinctive retinopathy that can result in blindness is a recognized feature of JPD [10, 23]. Both subjects had evidence of early retinopathy (angioid streaks) by their early thirties. Given that denosumab acts in a similar manner to OPG, one hope with its use is that retinopathy may be prevented or progression delayed. The experience of subject 1 suggests this hope may not be justified, as she lost sight in one eye after 11 years’ treatment. The cause was macular edema, but she had an excellent response to intravitreous therapy.

Denosumab may not be so effective in cases of JPD associated with rarer mutations, such as in the *SP7* gene [4], because the RANK/RANKL/OPG system is not directly affected in these cases.

In conclusion, long-term denosumab administration in adults with JPD, who had received previous bisphosphonate treatment, was safe and effective in terms of the skeletal disease, but it may not prevent the emergence of retinopathy. The limited experience to date suggests that caution is needed if denosumab is given to children with JPD who have very high rates of bone turnover.

## Data Availability

Data are available by the corresponding author upon reasonable request.
